# HPB News

**DOI:** 10.1155/1991/43038

**Published:** 1991


					HPB Surgery, 1991, Vol. 5, p. 77
Reprints available directly from the publisher
Photocopying permitted by license only
1991 Harwood Academic Publishers GmbH
Printed in the United Kingdom
HPB NEWS
BASILE AWARD TO TOM STARZL
The most prestigeous prize in Italian medicine, the Basile Award, was given to
Professor Tom Starzl at the occasion of the 2nd Congress of the Italian Chapter of
the World Association of Hepato-Pancreato-Biliary Surgery in Taormina in June,
1991. The prize was given to him for his enormous contributions to human liver
transplantation. The prize which is 50 000 USD was given to him in connection with
an honorary lecture given to the international audience.
77

				

